# Student Perceptions of a Hands-on Practicum to Supplement an Online eHealth Course

**DOI:** 10.2196/jmir.2029

**Published:** 2012-12-18

**Authors:** Sisira Edirippulige, Anthony C Smith, Nigel R Armfield, Mark Bensink, Richard Wootton

**Affiliations:** ^1^Centre for Online HealthThe University of QueenslandBrisbaneAustralia; ^2^Queensland Children's Medical Research InstituteThe University of QueenslandBrisbaneAustralia; ^3^Fred Hutchinson Cancer Research CenterSeattle, WAUnited States; ^4^Norwegian Centre for Integrated Care and TelemedicineTromsoNorway

**Keywords:** Telemedicine, Remote consultation, Education and training, curriculum

## Abstract

**Background:**

Since 2000, the Centre for Online Health (COH) at The University of Queensland has offered a range of online eHealth courses at the undergraduate and postgraduate level. While online learning has a number of advantages, in some domains, it can present some challenges to the development of practical skills and experience.

**Objective:**

To assess students’ perceptions of the value of an eHealth practicum.

**Methods:**

To supplement our online learning program, we introduced an eHealth practicum component that aimed to expose students to a range of clinically relevant learning experiences. Subsequently, by means of a questionnaire, student perceptions of the practicum were assessed.

**Results:**

Over two semesters, a total of 66 students participated in the eHealth practicum, and questionnaire responses were very positive. The majority of students agreed that the practicum allowed them to gain necessary skills in eHealth applications (59%) and provided them with an opportunity to explore ways of using different eHealth tools for the delivery of health care at a distance (62%).

**Conclusions:**

The study shows that a practical component in eHealth teaching was well received by students. While online teaching is appropriate for providing knowledge, the opportunity to develop practical skills may encourage students to use eHealth techniques in their future practices.

## Introduction

### Background

eHealth is an umbrella term that describes the use of information communication technologies (ICT) in health care. eHealth encompasses the areas of telehealth and telemedicine, which provide health services at a distance [1]. Evidence is emerging that eHealth can be useful in clinical, administrative, educational, and research settings [2-5].

One of the barriers often attributed to the slow uptake of eHealth is the lack of appropriate training and education. Research has shown that health professionals have limited knowledge and skills necessary for the effective use of eHealth applications [6,7]. A study conducted at a tertiary hospital in Australia showed that, although nurses were keen to use eHealth, they had limited opportunity to acquire appropriate knowledge and practical skills [8,9].

###  Theoretical Framework

There are a range of published resources that qualify the role of higher education in providing key skills for professional life [10-12]. Education research has highlighted the problems relating to a skills-mismatch between university graduates and the demand of the labor market [13-15].

Research suggests that skills acquired through university education have an effect on the performance of graduates in their professional life [16,17]. Evidence also suggests that there is a direct correlation between the core skills acquired through university education and the subsequent employability of university graduates [18,19]. Research has shown that university graduates seek jobs in the labor market where their skills are optimally rewarded [20,21].

Various educational reforms around the world are testimony to the attempts to address these issues [22-24]. The Dearing Report in the United Kingdom in 1997 emphasized the need for universities to “equip graduates with skills appropriate for work”. The report also recommended universities closely look at the needs of employers while developing curriculum [24].

Education research relating to skills development within higher education suggests that skills and competencies required for professional life vary widely across different disciplines and professions [25-27]. Therefore it is important to identify key skills and competencies that need to be addressed when developing learning activities. This argument is central to the pedagogical concept of the “alignment of learning objectives with learning outcomes” [28]. Research shows that learning activities must be designed with a thorough consideration of learning outcomes. Ramsden (2003) emphasized the importance of considering the views of various stakeholders to identify learning outcomes that must be met by the educational activities. Ramsden recommended gaining the opinions of students, lecturers, and employers as a valuable source of information in this regard [25].

Depending on the expected outcomes, the learning methods and activities may vary. Pedagogical research relating to learning methods is a dynamic area. Problem-based learning, work-based simulation, and experiential learning are some of the established educational methods. Changes in the learning environment, intended outcomes, and introduction of new technologies have prompted curriculum developers to seek new teaching and learning methods [29-31].

**S**ince 2000, the Centre for Online Health (COH) at The University of Queensland, Brisbane, Australia, has offered a range of eHealth courses at undergraduate and postgraduate levels. While the courses have been successfully delivered in an online mode, a survey of students (n=47) revealed that the majority (n=40, 86%) did not have the technological know-how or practical expertise to establish or to use eHealth systems. However, most of the students (n=37, 79%) believed that relevant practical skills would be useful in their future practice, and most students (n=41, 88%) also indicated that they would be keen to attend an in-person practicum to acquire such skills.

A curriculum development team was established within the COH. The team consisted of eHealth teaching staff and COH telehealth researchers. The team held regular meetings to discuss issues relating to the content and delivery mode of the practical sessions (practicum). In the process of developing the eHealth practicum, extensive consultation was conducted with relevant stakeholders. The organizations such as the Queensland Health Statewide Telehealth Services and the Australian Defence Force (ADF) were included as key stakeholders because they have a specific interest in eHealth. The staff members of these organizations are regular participants in eHealth courses.

The practicum was designed to supplement the online learning program. It aimed to offer students an opportunity to identify the relevance of eHealth in clinical practice, gain knowledge about technical options available for clinical eHealth, compare and contrast eHealth and conventional clinical communication, and develop communication skills relevant for eHealth. The practicum had four specific objectives: (1) to give students practical skills in various eHealth applications, (2) to allow students to explore ways of using different technologies in the delivery of health care, (3) to raise students’ awareness of the potential and limitations of the use of technology in health care, and (4) to allow students to reflect on key areas previously covered in their eHealth course.

## Methods

A 1-day eHealth practicum was introduced into the undergraduate eHealth course. It was delivered at the COH, based at the Royal Children’s Hospital, Brisbane, Queensland, and was an assessed component of the course. The practicum included a range of activities including introductory oral presentations, 4 hands-on practical exercises, observation of clinical teleconsultations, and visits to relevant sites within the hospital.

For quality assurance purposes, the university routinely evaluates all teaching programs. This study used routine student evaluation data. Completion of the evaluation form by participants was voluntary and anonymous.

### Structure of the Day

#### Orientation

The day commenced with an oral presentation on aspects of clinical eHealth followed by an overview of the activities of the day.

#### Practical Activities

Subsequently, the students cycled through 4 hands-on practical activities that were designed to provide a variety of relevant experiences for the students. The activities were based on active research projects within the COH, and learning was guided by research staff. The activities required students to complete practical tasks, including the role play of clinical interactions. Following each practical activity, the students were provided with a short debrief by the supervising member of the research staff. This allowed the opportunity to reinforce learning objectives, for students to reflect on the activity, and for them to ask questions.

#### Observation of Clinical Teleconsultations

While present in the COH, students were able to observe clinical teleconsultations between the tertiary hospital and referring hospitals. During these sessions, students could observe the nature of the clinical interactions between the tertiary specialists and the clinicians, patients, and families at the referring hospitals. The types of specialty teleconsultations observed included neurology, endocrinology, and burns depending on clinical activity within the hospital on the day of the practicum.

#### Site Visits

In addition to the activities conducted within the COH, students visited two other relevant facilities within the hospital campus. First, the students visited the Queensland Health Skills Development Centre (SDC), which is a specialist facility equipped with leading-edge simulation technology for the education and training of health professionals. In the SDC, students were given an overview of a range of technologies used including simulation applications. Second, the students visited the medical imaging department where they were provided with demonstrations of the use of a large-scale picture archiving and communication system (PACS).

### Description of the Hands-on Practical Activities

#### Activity 1: Standards-Based Videoconferencing— Familiarization With Equipment and Simulated Clinical Consultation

The rationale for the activity was to: (1) provide students with direct exposure to videoconferencing equipment, including identification of components and configuration of a working system, (2) provide students with an understanding of the benefits and limitations of clinical consultation using videoconferencing, and (3) compare and contrast the limitations and benefits with those experienced in Activities 2 and 3.

**Figure 1:**
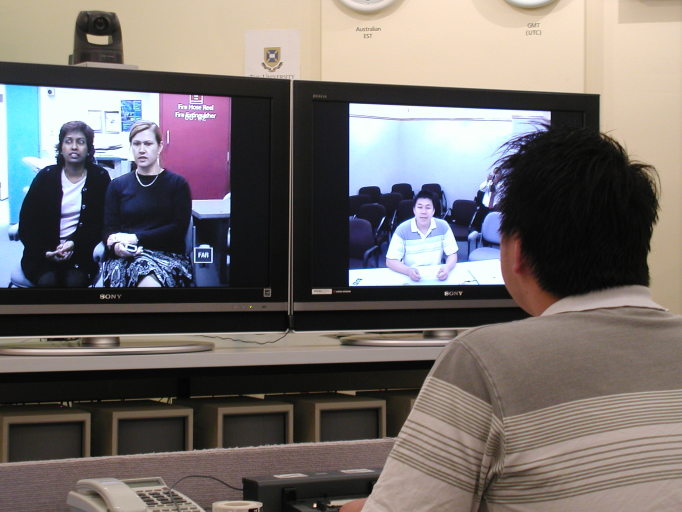
Students using standards-based videoconferencing.

Students were introduced to a fully configured telemedicine room, and key features such as videoconferencing and ancillary equipment, room lighting, and soundproofing were explained and demonstrated. Subsequently, students were provided with a kit of equipment and guidance to configure a basic videoconferencing endpoint. On completion of this task, students used the equipment to place a video call to a distant site (in this case, to their student colleagues in an adjacent room). Using the video link, students participated in a simulated clinical consultation which was designed to demonstrate the quality of audio, images, and movement. Students were guided to make calls at different data rates (128 kbit/s, 256 kbit/s, and 384 kbit/s) and to observe the changes in image and audio fidelity. During the consultation, students also used peripheral equipment consisting of a video document camera and a video-otoscope. Figure 1 shows students using videoconferencing equipment.

#### Activity 2: Internet and Webcam-Based Video Consultation

The rationale for this activity was to: (1) provide students with direct exposure to the benefits and limitations of clinical consultation using Internet and webcam-based video communication, and (2) compare and contrast the benefits and limitations with those experienced in Activities 1 and 3.

**Figure 2:**
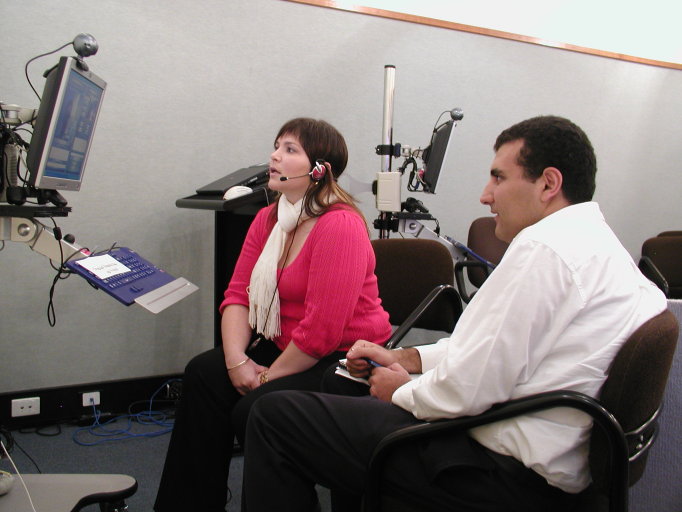
Students role playing home-telehealth using a webcam based system.

Students were introduced to a webcam-based palliative care telehealth application (see Figure 2). Subsequently, students participated in a room-to-room role play teleconsultation. The role play was designed to simulate a low-cost approach to linking clinicians at a tertiary hospital with a patient at home. As with Activities 1 and 3, students were guided to assess the quality of the audio, clarity of images, and reproduction of movement. In this activity, a single low-bit rate link was used to simulate that typically available to the home.

#### Activity 3: Simulated Consultation Using a Custom-Built Neonatal Intensive Care Teleconsultation System

The rationale for the activity was to: (1) provide students with direct exposure to the benefits and limitations of a teleconsultation system that had been highly customized to a particular clinical problem, and (2) compare and contrast the benefits and limitations with the generic communication approaches experienced in Activities 1 and 2.

**Figure 3:**
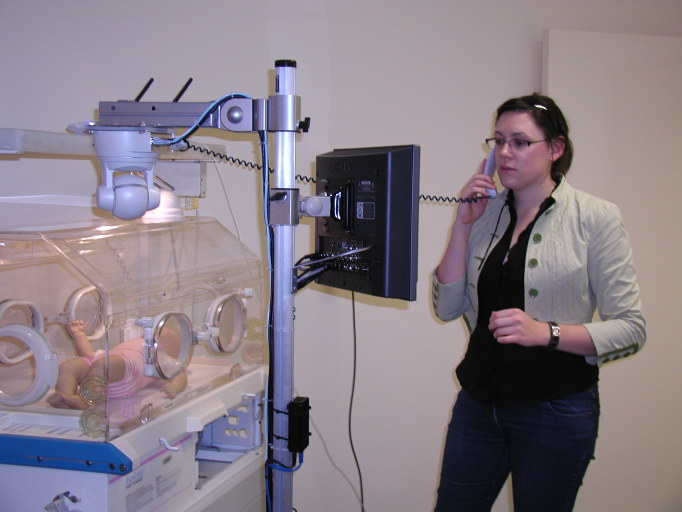
Simulated consultation using a specialized telemedicine system.

Students were introduced to a specialized telemedicine system that had been designed for providing remote specialist advice in neonatal care (see Figure 3). The highly customized system consisted of two parts: (1) a mobile wireless trolley with two cameras and a high degree of remote control for the referring hospital, and (2) a personal computer-based system for the specialist at the tertiary hospital. Using this system, students conducted a consultation that simulated a link between a tertiary intensive care nursery and a referring hospital with a sick newborn infant. The consultation included the viewing of high-quality live images of an infant mannequin, X-ray images, and the observation of a simulated patient monitor.

#### Activity 4: Techniques of Digital Photography for eHealth

The rationale for the activity was to provide students with knowledge and experience in taking clinically useful digital still images.

**Figure 4:**
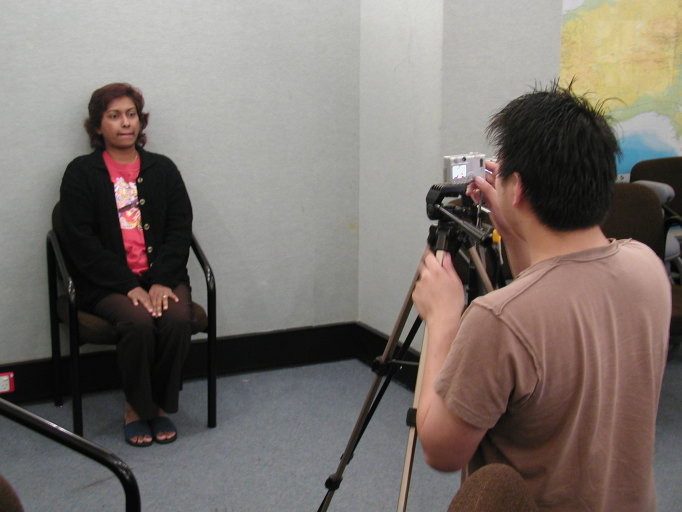
Students practicing aspects of clinical photography.

In contrast to the interactive nature of the previous 3 activities, this task was of relevance to store-and-forward telehealth. Clinical disciplines relevant to this task may include dermatology, wound care, or burns. Students were introduced to the features of standard digital cameras and techniques to capture clinically useful digital images. Guided through a series of 5 photography exercises, students experimented with aspects of focus, resolution, compression, lighting, angle, and background (see Figure 4). Following capture of images, students were required to upload and transfer the images by email to the lecturer for assessment.

### Evaluation

The aim of the evaluation was to assess students’ perceptions of the practicum, and hence a qualitative approach was taken. An evaluation questionnaire was designed and provided to all students on the day of the practicum. Students were requested to complete and return the questionnaire after the academic activities of the day had been completed. The questionnaire posed questions on certain themes as described in Table 1.

**Table 1:** Themes and assessment scale.

Theme	Response
The extent to which practicum objectives had been met (5 questions)	5-item Likert scale with neutral center value
Overall relevance of the practicum to the eHealth course (1 question)	5-item Likert scale with neutral center value
Structure and delivery (5 questions)	5-item Likert scale with neutral center value
Duration of the practicum (1 question)	3-item scale where center value represented “ideal”
Appropriateness of the level of the material presented (1 question)	3-item scale where center value represented “ideal”. Free text response was allowed for additional comments.
Overall assessment	Ordinal scale range 1 to 5 with 5 representing the highest level of satisfaction

To gain further insight, students were asked to provide free text responses for the following 5 themes:

1. Whether the practicum met the student’s expectations
2. The perceived strengths of the practicum that should be retained in the future
3. Suggestions for improvement
4. Participant’s area of work (eg, nursing, physiotherapy, information technology)
5. Speculation of how the skills gained may be useful in the student’s future practice

## Results

A total of 66 students took part in the eHealth practicum during 2010 (semester 1, 37 students and semester 2, 29 students). These undergraduate students had a health background. Students majoring in occupational therapy (n=29, 44%), physiotherapy (n=20, 30%), speech therapy (n=10, 15%), psychology (n=4, 6%), and bachelor of health sciences (n=3, 5%) participated in the practicum. A summary of questions and the results are shown in Table 2.

The majority of students (n=59, 89%) agreed that they obtained necessary practical skills in various eHealth applications. Similarly, the majority of students agreed that the practicum provided them an opportunity to explore ways of using different technologies in delivering health care while making them aware of both the potential and the limitations of eHealth. Students thought that the practicum was an opportunity to reflect on key areas covered in the eHealth course.

Students also agreed that the practicum was aligned with overall course objectives and provided skills relevant to eHealth practice; 63 students (95%) agreed that the practicum was totally relevant to the course.

When asked if the eHealth practicum met their expectations, 37 (56%) students noted that the practicum exceeded their expectations, indicating that the practicum was informative, enjoyable, interesting, and useful.

There was a mixed response to the structure and some features of the practicum. The majority of students (n=62, 94%) agreed that the practicum was structured well and the activities were designed to develop practical skills (see Table 3). The students thought that the instructors provided useful guidance during the practicum. However, students thought that lectures provided at the beginning of the practicum day were not useful. Some suggested that those lectures were a repetition of the material provided in the online course. Similarly, they suggested that the quality of hand-outs should be improved.

In addition, 57 students (86%) agreed that the duration of the practicum was appropriate. Only 6 (9%) students felt that the practicum was too long. The majority of students (n=59, 89%) also agreed that the practicum was pitched at a level appropriate to their knowledge and technical ability.

When asked whether the hands-on skills acquired from this practicum would be useful in their future practice, 60 students (90%) noted that the skills would be extremely useful.

Multimedia Appendix 1 shows extracts from the written comments provided in the students’ evaluation forms in relation to the usefulness of the skills acquired through the practicum.

**Table 2:** Content of the eHealth practicum.

Objectives: This practicum enables you to...	Responses (%)	Strongly disagree (%)	Disagree (%)	Uncertain (%)	Agree (%)
1. obtain necessary hands-on skills in various eHealth applications	66 (100)	0	0	7 (11)	59 (89)
2. explore ways of using different technology in the delivery of health care	66 (100)	0	0	4 (6)	62 (94)
3. raise awareness of the potential and limitations of the use of technology in health care	66 (100)	0	0	11 (17)	55 (83)
4. reflect on key areas covered in your eHealth course	66 (100)	0	0	3 (5)	63 (95)

**Table 3:** Summary of responses related to the organization of the practicum.

Practicum	Responses (%)	Strongly disagree (%)	Disagree (%)	Uncertain (%)	Agree (%)
1. This practicum was appropriately structured.	66 (100)	0	0	4 (6)	62 (94)
2. The activities were designed to develop necessary skills.	66 (100)	0	0	0	66 (100)
3. The oral presentations were useful.	66 (100)	0	0	30 (45)	36 (55)
4. Hand-outs were useful.	66 (100)	0	0	45 (68)	21 (32)
5. Instructors’ guidance was adequate.	66 (100)	0	0	10 (15)	56 (85)

## Discussion

E-learning (online learning) offers many advantages. Evidence shows the potential of e-learning to facilitate better access, better learning outcomes, and cost savings in medical and health education. However, the suitability of e-learning methods depends on the educational outcomes intended.

eHealth is an emerging discipline. While evidence for the benefits of eHealth is growing, the use of eHealth in mainstream health care is still limited. Among other reasons, the lack of appropriate education and training has been cited. eHealth involves not only the use of new technologies in health care settings but also new ways of practicing health care. eHealth changes the dynamics of communication and interaction of parties involved. While a conceptual understanding of these changes is important, the practice of eHealth requires specific practical skills and competencies. E-learning may not always be capable of facilitating the development of such skills and competencies. In some cases, traditional experiential learning methods such as a work-based practicum may be better suited to provide such skills and competencies.

In this study, a practicum was incorporated into an eHealth course, which was previously offered entirely online. The practicum was carefully designed to develop a set of skills that would help students to practice eHealth in their future professions. The majority of students who attended the practicum had a health sciences background, and they were planning to work in rural and remote areas after their graduation.

The activities in the practicum were based primarily on the COH projects [32,33]. A range of activities were selected to offer students the opportunity to obtain experience using different types of eHealth applications (eg, real time and store-and-forward) and various equipment (eg, commercial, web-based, and purpose-built videoconference systems). While students had learned about eHealth applications in the course, the practicum was an opportunity to experience and reflect on key areas covered by the course. The results of the study showed that the practicum was well received by the students. Students agreed that the practicum enabled them to obtain necessary skills in various eHealth applications. Students noted that the practicum provided the opportunity to explore ways of using different technologies in delivering health care at distance.

The practicum not only engaged students in establishing eHealth units and set up communication links but also provided the opportunity to observe how eHealth is practiced for clinical purposes. Students were able to attend some eHealth sessions where clinicians provided consultations to remote patients. Students also had the opportunity to ask clinicians questions after these sessions. The observation of actual eHealth interactions complemented by scenario-based role play allowed students to appreciate both the potential and the challenges of using eHealth in clinical settings. In their written comments, students noted that the practicum was useful for them to reflect on the relevance of eHealth activities in their future practices.

The reported high student satisfaction can be explained by the novelty of the activities and the relevance of skills and competencies developed during the practicum. In the design of the practicum, the curriculum team paid particular attention to the views of various stakeholders including students, lecturers, and the potential employers. Close communication with the stakeholders offered the opportunity to understand the expectations of relevant parties.

Compared with ratings for the simulation activities, student ratings for the didactic lectures that took place on the day were low. This finding, which occurred early in the program, prompted the educators to refocus the structure of the practicum entirely on simulation activities. This reinforces the suitability of e-learning for didactic delivery and the in-person practicum approach for the development of practical skills. However, it may also be the case that relative to the practical activities, the lectures were simply less interesting to the students.

To the best of the authors’ knowledge, this is the first study in the literature that describes an attempt to design, deliver, and evaluate a practicum to supplement an online eHealth course. The integration of eHealth into mainstream health care requires systematic education and training of current and future health professionals. Therefore, research into eHealth education and training must be given more attention. eHealth education must focus not only on the provision of knowledge about eHealth applications but also the development of relevant practical skills and competencies that will be useful in practice. This study shows the significance of work-based experiential learning in developing skills in eHealth.

### Conclusions

This study showed that the opportunity to participate in an eHealth practicum as a part of an undergraduate online course was highly valued by students. Having practical skills may encourage clinicians to use eHealth in their clinical practice. Therefore, education and training in eHealth must incorporate the development of such skills and competencies. The study showed the value of a blended learning approach, using e-learning to teach theoretical aspects and experiential learning for students to develop practical skills. Given the opportunity, students may use knowledge and skills relating to eHealth in their future practices. The emphasis on education and training of eHealth may be an important step to address the slow uptake of eHealth in the workplace. Future studies must formally assess the effectiveness of eHealth education and training.

## Multimedia Appendix 1

Students' written comments.
