# Supplementary material for: Student Perceptions of a Hands-on Practicum to Supplement an Online eHealth Course
Source: J Med Internet Res. 2012 Dec 18;14(6):e182. doi: 10.2196/jmir.2029 (PMC3799484; doi:10.2196/jmir.2029)
Supplement: Supplementary file 1 [file jmir_v14i6e182_app1.pdf]

Multimedia Appendix 1. Students' written comments.

Skills acquired

- I learned how to set up a videoconference.
- Knowing the differential use of bandwidth is useful.
- I learned how to take clinically appropriate pictures.

Benefits as a health professional

- I would like to use these skills when I become a doctor. I realized how ICT can be used for my consultations.
- My teaching profession can benefit from some of these skills.
- I can use these skills in my profession as a clinical pharmacist working in rural areas.
- I learned how to use technology, particularly videoconferencing, for clinical practice.
- Possibly I can help doctors and patients from developing countries.
- As a speech pathology specialist in rural/remote location, eHealth techniques can be very useful.
- eHealth skills will be useful as a physiotherapy professional.
- This will be useful in home monitoring of rehab programs.
- As a medical professional, eHealth tools will be very useful in the future.
- eHealth can be useful in maintaining contacts with clinicians.

Benefits for patients

- The skills acquired in the practicum will be helpful to assist my patients to reduce travel and save money.
- These skills can help patients, especially those who do not have access to allied health services in rural areas.
- I will use videoconferencing in my psychology advice to provide services to remote patients.
- I realized that simple technologies such as webcams can be used to consult patients in regional areas (where mental health may not be accessible).
- eHealth can be a useful tool in aged care.

Attitude change

- The practicum helped me become more aware of the direction technology is shaping health care.
- Simple webcams can be a useful tool to help patients.
